# Infectious Spleen and Kidney Necrosis Virus Triggers Ferroptosis in CPB Cells to Enhance Virus Replication

**DOI:** 10.3390/v17050713

**Published:** 2025-05-16

**Authors:** Qiushuang Zhang, Ouqin Chang, Qiang Lin, Hongru Liang, Yinjie Niu, Xia Luo, Baofu Ma, Ningqiu Li, Xiaozhe Fu

**Affiliations:** 1 College of Fisheries and Life Science, Shanghai Ocean University, Shanghai 201306, China; 2 Pearl River Fisheries Research Institute, Chinese Academy of Fishery Sciences, Key Laboratory of Fishery Drug Development, Ministry of Agriculture and Rural Affairs, Guangdong Provincial Key Laboratory of Aquatic Animal Immunology and Sustainable Aquaculture, Guangzhou 510380, China

**Keywords:** ferroptosis, ISKNV, viral replication, GPx4, ACSL4

## Abstract

The role of ferroptosis—a novel iron-dependent programmed cell death pathway—in infectious spleen and kidney necrosis virus (ISKNV) infection remains poorly understood. Here, we demonstrate that ISKNV infection induces ferroptosis in CPB cells. Following ISKNV challenge, CPB cells exhibited hallmark morphological alterations including mitochondrial shrinkage, increased membrane density, and cristae reduction. Biochemical assays confirmed significant time-dependent elevations in ferroptosis markers: malondialdehyde (MDA, 1.7-fold), reactive oxygen species (ROS, 3.14-fold), and ferrous iron (Fe^2+^, 1.42-fold) compared to controls (*p* < 0.05). Mechanistic studies revealed that ISKNV downregulated glutathione peroxidase 4 (GPx4) while upregulating acyl-CoA synthetase long-chain family member 4 (ACSL4), as validated by quantitative real-time PCR (qRT-PCR) and immunoblotting. Ferroptosis induction with erastin enhanced ISKNV replication, whereas inhibition with liproxstatin-1 suppressed viral yield. These findings establish that ISKNV exploits ferroptosis to facilitate its replication, and pharmacological blockade of this pathway significantly suppresses viral propagation, providing a new strategy and intervention approach for controlling ISKNV infection.

## 1. Introduction

The rapid expansion of *Siniperca chuatsi* (mandarin fish) aquaculture in China has been accompanied by increasing outbreaks of viral diseases, causing substantial economic losses. Among them, infectious spleen and kidney necrosis virus (ISKNV) is one of the most common outbreak diseases in *Siniperca chuatsi* [1]. ISKNV is a double-stranded DNA virus, belonging to the Megalocytivirus genus of the Iridoviridae family, with 111,362 bp DNA genome encoding 124 potential ORFs [2]. ISKNV can infect more than 50 fish species and has strong pathogenicity [3]. Under suitable temperatures, its mortality rate can reach 100% [4]. A Chinese perch brain (CPB) cell line infected with ISKNV showed characteristic cytopathic effects, including cell rounding, enlargement, and eventual detachment, consistent with megalocytivirus pathogenesis [5].

Ferroptosis has emerged as a distinct form of regulated cell death characterized by iron-dependent lipid peroxidation and reactive oxygen species (ROS) accumulation [6]. Unlike apoptosis, necrosis, or autophagy, ferroptosis exhibits unique morphological features, particularly mitochondrial shrinkage, increased membrane density, and a reduction in cristae [7,8,9]. Biochemically, ferroptosis induces iron overload, reactive oxygen species (ROS) accumulation, and lipid peroxidation through regulating iron metabolism, lipid metabolism, glutamine metabolism, and the system Xc-/glutathione peroxidase 4 (GPx4) pathway [10,11].

Growing evidence suggests that viral infections can disrupt cellular homeostasis by modulating metabolic pathways and cell death mechanisms [12,13,14]. Several viruses, including Newcastle disease virus (NDV) [12], human adenovirus type 7 (HAdV-7) [15], and novel duck orthoreovirus (N-DRV) [16], have been shown to induce ferroptosis through distinct mechanisms, such as iron accumulation, ROS generation, and GPx4 inhibition. However, whether ISKNV exploits ferroptosis to facilitate its replication remains unknown.

In this study, we demonstrate that ISKNV infection triggers ferroptosis in CPB cells by inducing mitochondrial dysfunction, lipid peroxidation, and iron accumulation, as well as downregulating GPx4 and upregulating ACSL4. Furthermore, pharmacological induction of ferroptosis enhanced ISKNV replication, whereas its inhibition suppressed viral propagation. These findings establish ferroptosis as a new mechanism in ISKNV infection and highlight its potential as a therapeutic target for ISKNV control in mandarin fish aquaculture.

## 2. Materials and Methods

### 2.1. Cells, Virus, and Main Reagents

The Chinese perch brain cell line (CPB) [17], ISKNV-QY strain [18], and ISKNV-MCP monoclonal antibody [19] were kept in our laboratory. Rabbit monoclonal anti-SLC7A11 was purchased from Abmart (Shanghai, China). Rabbit monoclonal anti-GPx4, FTH1, SLC40A1, ACSL4, and rabbit monoclonal anti-β-actin were purchased from Proteintech (Wuhan, China). Erastin and liproxstatin-1 were purchased from Macklin (Shanghai, China).

### 2.2. Cell Culture and Virus Infection

CPB cells were cultured in Leibovitz’s L-15 medium supplemented with 8% fetal bovine serum (FBS) at 28 °C in a CO_2_-free incubator and passaged at 1:3 ratios every 2–3 days upon reaching 80–90% confluence. For infection, semi-confluent monolayers (70% confluence) were washed with PBS and inoculated with ISKNV diluted 1:100 in serum-free L-15 medium for 2 h at 28 °C, after which the inoculum was replaced with maintenance medium (L-15 + 2% FBS). When 90% cytopathic effect was observed, the virus was harvested by freeze–thaw cycling of cell lysates and supernatants, with aliquots stored at −80 °C. Viral titers were determined by TCID_50_ assay on CPB cells. Briefly, the viral suspension was serially diluted in L-15 medium at gradients ranging from 10^−1^ to 10^−10^. Each dilution was added to a 96-well plate (100 μL/well), with eight replicates per dilution. Untreated cells served as the control group. CPE was monitored daily, and data were recorded for two weeks. The Reed–Muench two-way method was used to calculate the final results [20].

### 2.3. Integrated Multi-Omics Analysis of Ferroptosis During ISKNV Infection

Building upon our previous transcriptomic [21] and metabolomic [22] profiling of ISKNV-infected CPB cells, this study specifically investigated ferroptosis-related pathways through targeted multi-omics analysis. Gene expression dynamics of core ferroptosis regulators were quantified at 24 and 72 h post-infection (hpi) using normalized RNA-seq data reanalyzed with Origin 2021 (v9.8), with significance thresholds set at |log2FC| ≥ 1 and FDR < 0.05. Parallel LC-MS/MS-based metabolomics measured time-dependent changes in polyunsaturated fatty acids and phospholipids, critical substrates for lipid peroxidation in ferroptosis.

### 2.4. Cytotoxicity Assessment of Ferroptosis Modulators Using CCK-8 Assay

To establish the non-toxic working concentrations of ferroptosis inducer (erastin) and inhibitor (liproxstatin-1) for subsequent experiments, CPB cell viability was evaluated using the Cell Counting Kit-8 (CCK-8) assay. Briefly, cells were seeded in 96-well plates (2 × 10^5^ cells/well in 100 μL of L-15 medium with 8% FBS) and allowed to adhere for 24 h at 28 °C under CO_2_-free conditions. Cells were then treated with serial dilutions (1–500 μM) of erastin, liproxstatin-1, or equivalent concentrations of the vehicle control (DMSO) for 72 h. Following treatment, cells were incubated with CCK-8 reagent (10 μL) diluted in 100 μL of L-15 medium with 2% FBS for 4 h at 28 °C. Absorbance was measured at 450 nm using a microplate reader (BioTek Synergy H1, BioTek, VT, USA).

### 2.5. Ultrastructural Analysis by Transmission Electron Microscopy (TEM)

For TEM examination of ISKNV-induced cellular alterations, CPB cells (5 × 10^5^ cells/mL) were seeded in T_25_ flasks and infected with ISKNV at an MOI of 100. At 24, 48, and 72 hpi, cells were fixed with 2.5% glutaraldehyde in 0.1 M phosphate buffer (pH 7.4) for 24 h at 4 °C and then post-fixed in 0.1 M phosphate buffer containing 1% osmium tetroxide for 1 h. Ultrathin sections were stained with uranyl acetate–lead citrate and examined by a Philips CM10 electron microscope (Philips, Eindhoven, The Netherlands).

### 2.6. Fe^2+^, ROS, and MDA Detection

To assess ferroptosis induction during ISKNV infection, CPB cells (5 × 10^5^ cells/mL) were seeded in 6-well plates and infected with ISKNV at an MOI of 100. At 24, 48, and 72 hpi, cells treated with erastin or liproxstatin-1 were harvested for quantification of three key ferroptosis markers. The Fe^2+^ content of the cells was detected by laser scanning confocal microscopy and a microplate reader using the fluorescent probe FerroOrange (Elabscience, E-BC-F101, Wuhan, China). The ROS content of the cells was detected by laser scanning confocal microscopy and a microplate reader using the fluorescent probe DCFH-DA (Beyotime, S0033S, Shanghai, China). MDA level was detected by a microplate reader using the Cellular Malondialdehyde Determination Kit (Jiancheng Bioengineering Institute, A003-4-1, Nanjing, China).

### 2.7. RNA Extraction, Reverse Transcription, and Quantitative Real-Time PCR (qRT-PCR)

To assess ferroptosis gene expression during ISKNV infection, CPB cells (5 × 10^5^ cells/mL) were seeded in 12-well plates and infected with ISKNV at an MOI of 100. At 72 hpi, total RNA was extracted from ISKNV-infected and control CPB cells using the FastPure Complex Tissue/Cell Total RNA Isolation Kit (Vazyme, RC112-01, Nanjing, China). RNA quality and concentration were verified by spectrophotometry (A260/A280 ratio > 1.8) and agarose gel electrophoresis. First-strand cDNA was synthesized from 5 μg of total RNA using One-Step gDNA Removal and cDNA Synthesis SuperMix (TransGen Biotech, AT311-03, Beijing, China). qRT-PCR was performed in triplicate using SYBR Green Master Mix (Vazyme, Q711-02, Nanjing, China) on a QuantStudio 6 Flex Real-Time PCR System (Applied Biosystems, New York, USA) under the following conditions: 95 °C for 30 s; 40 cycles of 95 °C for 5 s; and 60 °C for 30 s. The reaction volume of SYBR Green was 20 μL, including 10 μL 2× SYBR Premix Enzyme containing ROX, 0.5 μL each of forward and reverse primer (10 μM), and 7 μL of ddH_2_O and 2 μL of cDNA. The *18S rDNA* served as the endogenous control. Relative gene expression was calculated using the 2^−ΔΔCt^ method [23], with normalization to both *18S rDNA* and mock-infected controls. The used primers are listed in Table 1.

### 2.8. DNA Extraction and qPCR

To assess viral copies, CPB cells (5 × 10^5^ cells/mL) were seeded in 12-well plates, then treated with erastin or liproxstatin-1 either before or after ISKNV infection at an MOI of 100. Viral DNA was extracted from supernatants using proteinase K lysis (160 μg/mL final concentration) with incubation at 56 °C for 2 h followed by heat inactivation (95 °C, 10 min) and centrifugation (12,000× *g*, 3 min), while intracellular DNA was isolated using the FastPure^®^ Cell/Tissue DNA Isolation Mini Kit (Vazyme, DC102-01) according to the manufacturer’s protocol. TaqMan qPCR was performed on a QuantStudio 6 Flex system. The reaction system contained 10 μL of 2×Premix, 0.4 μL of primers, 0.4 μL of probe, 0.4 μL of ROX, 6.4 μL of ddH2O, and 2 μL of DNA. The procedure was carried out at 94 °C for 1 min, 94 °C for 10 s, and 60 °C for 30 s, for 40 cycles. The primers and probe are listed in Table 2. The copy numbers of ISKNV were calculated through comparison with the standard curve (Y = −3.314lgX + 41.48; Y refers to the Ct value and X refers to the copy number), as described previously.

### 2.9. Western Blot

Cells were collected and lysed in RIPA buffer with 1 mM PMSF. Protein samples were separated by 12.5% SDS-PAGE (80 V for 20 min, then 120 V for 60 min) and transferred to PVDF membranes using a semi-dry transfer system. Membranes were activated in methanol for 15 s, rinsed in deionized water for 2 min, and equilibrated in transfer buffer (25 mM Tris, 192 mM glycine, 20% methanol) for 1 min. Proteins were transferred at 150 V for 55 min with the membrane positioned above the gel to ensure proper orientation. Following transfer, membranes were blocked with 3% BSA in TBST (Tris-buffered saline with 0.1% Tween-20) for 3 h at room temperature. Primary antibody (SLC7A11 (1:2000), GPx4 (1:2000), FTH1 (1:2000), SLC40A1 (1:2000), ACSL4 (1:2000), and ISKNV-MCP (1:500)) incubation was performed overnight at 4 °C in blocking buffer, followed by three 5 min TBST washes. Membranes were then incubated with HRP-conjugated secondary antibody (1:10,000 dilution) for 1 h at room temperature. After additional washing (3 × 5 min TBST), protein bands were visualized using an enhanced chemiluminescence (ECL) substrate (Beyotime, Shanghai, China) and imaged with a ChemiDoc MP system (Bio-Rad, Shanghai, China).

### 2.10. Statistical Analysis

All data were analyzed using Origin 2021 (OriginLab Corporation), ImageJ-win64 (National Institutes of Health), and GraphPad Prism 8.0 (GraphPad Software). Group differences were compared using Student’s *t*-tests (for two groups), one-way ANOVA with Dunnett’s post hoc test (for multi-group comparisons against a single control), or two-way ANOVA with Sidak’s post hoc test (for multi-factor analyses). Statistical significance thresholds were defined as * *p* < 0.05, ** *p* < 0.01, and *** *p* < 0.001, with *p* > 0.05 considered not significant (NS).

## 3. Results

### 3.1. ISKNV Induces Ferroptosis Cell Death

**Figure 1:**
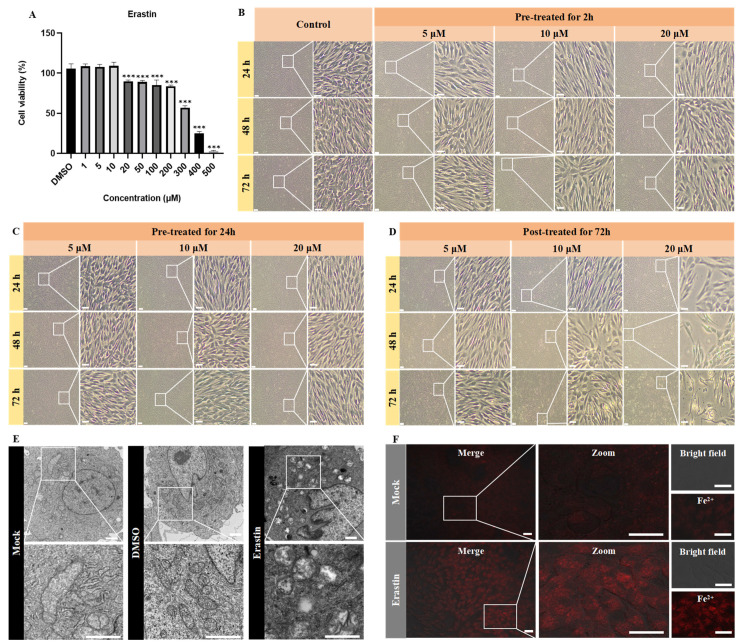
Ferroptosis induced with erastin in CPB cells. (**A**) Cytotoxicity of erastin in CPB cells, *** *p* < 0.001. (**B**–**D**) Morphological observation of CPB cells treated with erastin. CPB cells were pre-treated with erastin for 2 h and 24 h, or post-treated for 72 h with 5 μM, 10 μM, or 20 μM erastin, and then CPB morphology was observed at 24 h, 48 h, and 72 h. Scale bars = 100 μm. (**E**) Mitochondria morphological observation of CPB cells treated with erastin (10 μM, 48 h) by transmission electron microscopy. Scale bars = 1 μm. (**F**) Detection of Fe^2+^ levels in CPB cells after treatment with erastin (10 μM) for 48 h. Scale bars = 20 μm.

Although erastin is a well-characterized ferroptosis inducer in mammalian systems, its capacity to trigger ferroptosis in CPB cells remained unverified. Figure 1A demonstrates that erastin concentrations below 10 μM maintained CPB cell viability above 90%. Further comparative analysis of three erastin concentrations (5, 10, and 20 μM) administered via different treatment revealed distinct cellular responses. Pre-treatment 2 h or 24 h prior to assay showed no significant morphological alterations compared to untreated controls throughout the 24–72 h observation period (Figure 1B,C). In contrast, post-treatment, where CPB cells were continuously exposed to erastin throughout the entire duration of the experiment, the 5 μM group maintained normal morphology, and 20 μM erastin induced rapid cell detachment within 24 h, while 10 μM erastin elicited progressive cellular crumpling—a characteristic ferroptotic morphology—indicating post-treatment with 10 μM erastin was the optimal condition (Figure 1D). Upon the optimization of erastin treatment, the characteristic ferroptotic morphology, including mitochondrial cristae reduction, increased membrane density, and Fe^2+^ accumulation, was observed by TEM (Figure 1E) and confocal microscopy (Figure 1F). These results demonstrated that post-treatment with 10 μM erastin can induce ferroptosis in CPB cells.

**Figure 2:**
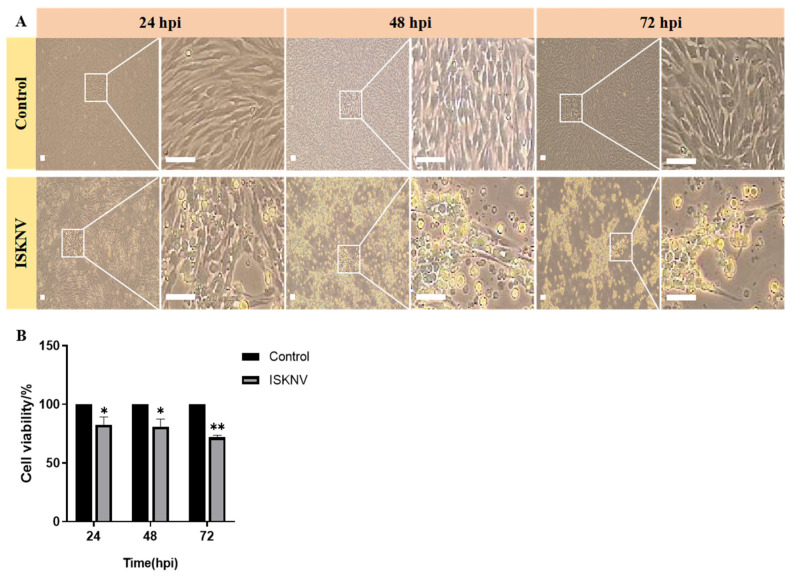
Changes in CPB cells in response to ISKNV. (**A**) Morphological changes in CPB cells infected with ISKNV (24–72 h). Scale bars = 100 μm. (**B**) Relative levels of cell viability post-ISKNV infection (24–72 h). * *p* < 0.05, ** *p* < 0.01.

**Figure 3:**
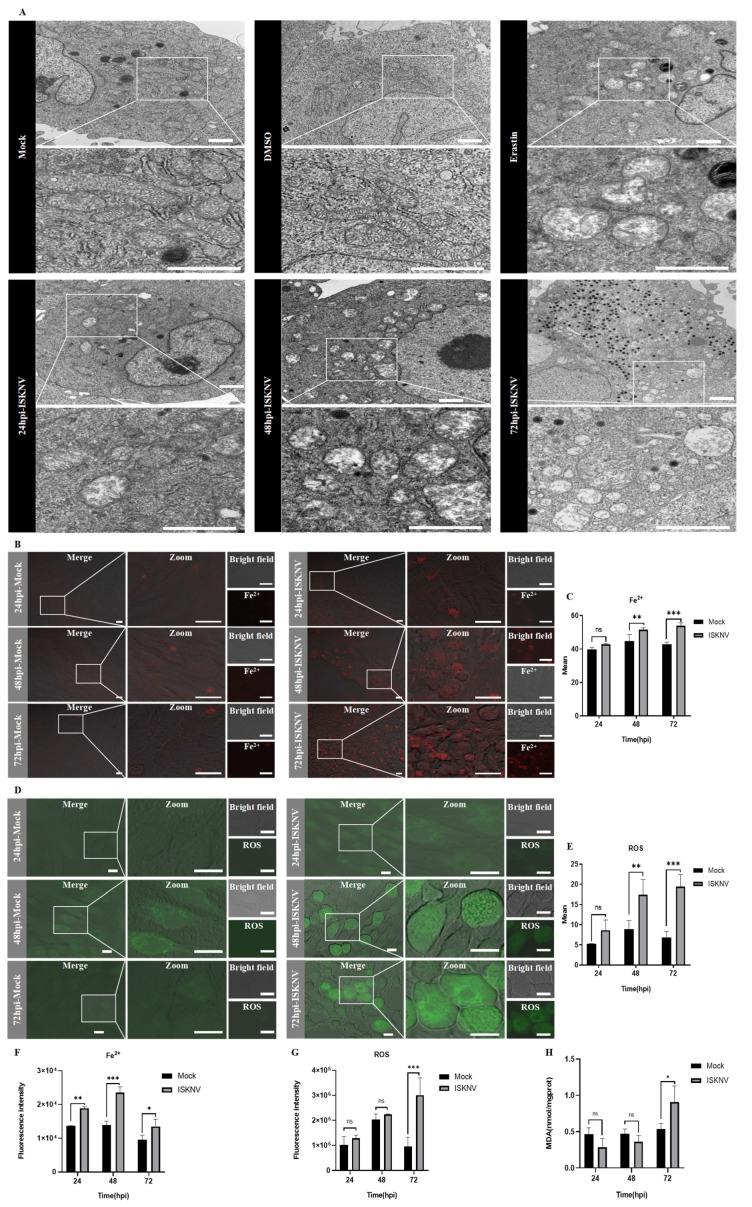
ISKNV infection induces ferroptosis in CPB cells. (**A**) Transmission electron microscopy of CPB cells treated with DMSO (72 h), erastin (10 μmol/L, 72 h), and ISKNV (100 MOI, 24 h, 48 h, 72 h). Scale bars = 1 μm. (**B**) Analysis of Fe^2+^ levels in CPB cells after treatment with ISKNV (100 MOI) using the fluorescent probe FerroOrange by laser scanning confocal microscopy. Scale bars = 20 μm. (**C**) Quantitative analysis of the mean fluorescence intensity of (**B**) using Image J. (**D**) Analysis of intracellular ROS levels using DCFH-DA staining, and laser scanning confocal microscopy of CPB cells treated with ISKNV (100 MOI) for 24–72 h. Scale bars = 10 μm. (**E**) Quantitative analysis of the mean fluorescence intensity of (**D**) using Image J. (**F**–**H**) Detection of Fe^2+^, ROS, and MDA levels in cell lysates treated with ISKNV (100 MOI) for 24–72 h by microplate reader. * *p* < 0.05, ** *p* < 0.01, and *** *p* < 0.001, with *p* > 0.05 considered not significant (ns).

Subsequently, ISKNV infection-induced ferroptosis in CPB cells was investigated. Firstly, ISKNV infection induces cell rounding and enlargement at 24–48 hpi followed by widespread detachment at 72 hpi, accompanied by progressive viability reduction (Figure 2). Furthermore, TEM observation revealed that mitochondrial shrinkage, increased membrane density, and cristae reduction were evident at 24 hpi; the cristae further decreased or disappeared at 48 hpi, and mitochondrial cristae loss with membrane rupture was observed at 72 hpi (Figure 3A). Confocal microscopy was used to observe the fluorescence of Fe^2+^ and ROS after ISKNV infection at 24 h, 48 h, and 72 h. The results showed ISKNV infection-dependent accumulation of Fe^2+^ (Figure 3B) and ROS (Figure 3D), and the average fluorescence intensity in the ISKNV group was higher than in the control group (Figure 3C,E). The levels of Fe^2+^, ROS, and MDA post-ISKNV infection for 24 h, 48 h, and 72 h were significantly higher than in the control group, with Fe^2+^ increasing from 1.38-fold at 24 hpi to 1.42-fold at 72 hpi (Figure 3F), ROS elevating from 1.26-fold (24 hpi) to 3.14-fold at 72 hpi (Figure 3G), and MDA rising 1.7-fold at 72 hpi (Figure 3H). The above results suggest that ISKNV infection induces ferroptosis in CPB cells.

### 3.2. Ferroptosis Increases ISKNV Replication in CPB Cells

**Figure 4:**
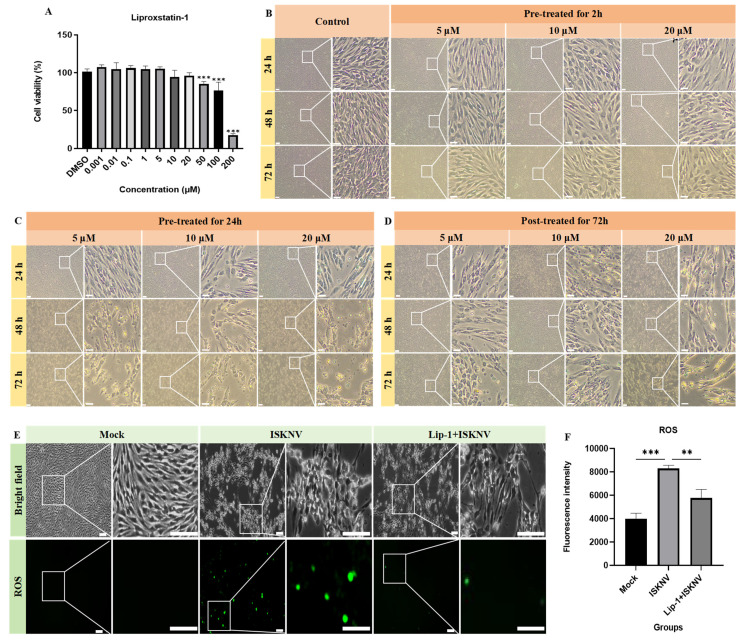
Liproxstatin-1 inhibited ferroptosis in CPB cells. (**A**) Cytotoxicity of liproxstatin-1 in CPB cells. (**B**–**D**) Morphological observation of CPB cells treated with liproxstatin-1. CPB cells were pre-treated with liproxstatin-1 for 2 h and 24 h, or post-treated with liproxstatin-1 for 72 h at 5 μm, 10 μm, and 20 μm concentration, and then CPB morphology was observed at 24 h, 48 h, and 72 h. Scale bars = 100 μm. (**E**,**F**) Detection of intracellular ROS levels treated with DMSO, ISKNV (100 MOI), and liproxstatin-1 + ISKNV for 24 h, using DCFDA staining and a microplate reader. Scale bars = 100 μm. ** *p* < 0.01, *** *p* < 0.001.

Liproxstatin-1 is a ferroptosis inhibitor, but its capacity to suppress ferroptosis in CPB cells remained unknown. Figure 4A demonstrates that liproxstatin-1 concentrations below 20 μM maintained CPB cell viability above 90%. Further comparative analysis of three liproxstatin-1 concentrations (5, 10, and 20 μM) administered via different treatments revealed distinct cellular responses. Pre-treatment for 24 h or post-treatment for 72 h induced significant cytotoxicity, such as cell aggregation and apoptotic morphology (Figure 4C,D). In contrast, pre-treatment for 2 h with 5–10 μM liproxstatin-1 maintained normal cellular morphology, while 20 μM treatment caused initial signs of cellular crumpling (Figure 4B). Therefore, 10 μM liproxstatin-1 with 2 h pre-treatment was selected for subsequent experiments. To further evaluate the inhibitory effect of liproxstatin-1 on ferroptosis, CPB cells were pre-treated with 10 μM liproxstatin-1 for 2 h prior to ISKNV infection (MOI = 100). The result showed liproxstatin-1 pre-treatment significantly decreased ISKNV-triggered ROS accumulation (Figure 4E). The above results validated the efficacy of the optimized liproxstatin-1 treatment protocol (10 μM, 2 h pre-treatment) in blocking ferroptotic signaling.

**Figure 5:**
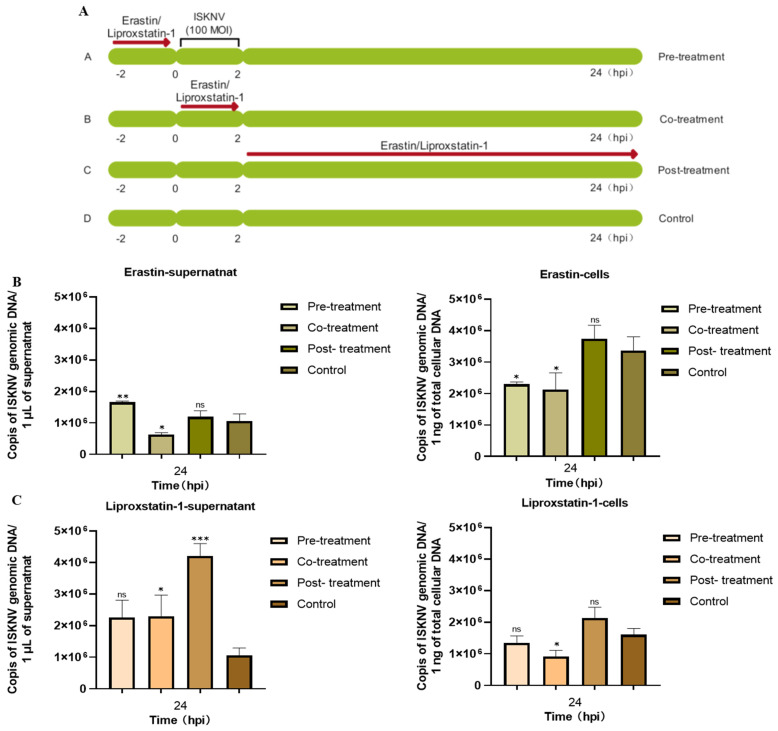
Detection of viral genomic DNA copies in CPB cells and supernatants after different treatments with erastin or liproxstatin-1. (**A**) Schematic diagram of erastin and liproxstatin-1 treatment methods. (**B**) CPB cells were pre-treated before ISKNV inoculation, co-treated with ISKNV inoculation, or post-treated after ISKNV inoculation with erastin (10 μmol/L). The DNA copies in the cells and supernatant were detected by qRT-PCR at 24 hpi. (**C**) CPB cells were pre-treated before ISKNV inoculation, co-treated with ISKNV inoculation, or post-treated after ISKNV inoculation with liproxstatin-1 (10 μmol/L). The DNA copies in the cells and supernatant were detected by qRT-PCR at 24 hpi. * *p* < 0.05, ** *p* < 0.01, and *** *p* < 0.001, with *p* > 0.05 considered not significant (ns).

To evaluate the potential effects of erastin and liproxstatin-1 on ISKNV entry processes, three treatment methods were used, including pre-treatment (2 h prior to infection), co-treatment (with viral inoculation), and post-treatment (2 h post-adsorption) (Figure 5A). qPCR results indicated that ISKNV DNA copy numbers following intracellular or extracellular post-treatment with erastin and pre-treatment with liproxstatin-1 had no significant difference compared to untreated controls (Figure 5B,C). These results demonstrated that post-treatment with erastin and pre-treatment with liproxstatin-1 had no effect on ISKNV adsorption and invasion and were used for the subsequent experiments.

**Figure 6:**
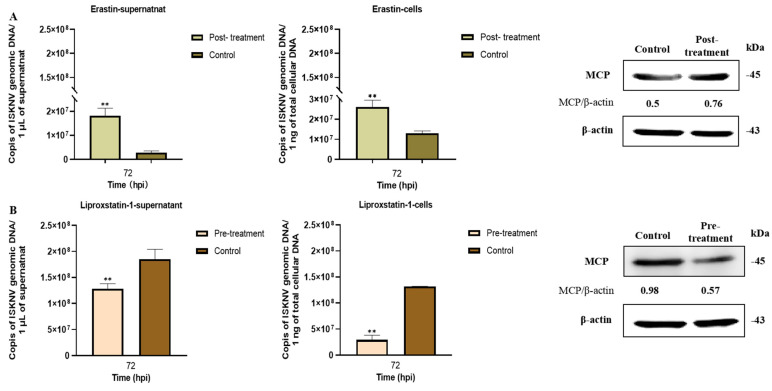
Effects of erastin and liproxstatin-1 on ISKNV replication. (**A**) Detection of viral genomic DNA copies and ISKNV MCP level after post-treatment with erastin (10 μM) at 72 hpi by qRT-PCR or Western blot. (**B**) Detection of viral genomic DNA copies and ISKNV MCP level after pre-treatment with liproxstatin-1 (10 μM) at 72 hpi by qRT-PCR or Western blot. ** *p* < 0.01.

Subsequently, the relationship between ferroptosis and ISKNV replication was assessed by qRT-PCR and Western blot analyses. The results demonstrated that erastin treatment significantly enhanced ISKNV replication, as evidenced by 6.4-fold and 2-fold increases in intracellular and extracellular viral DNA copies, respectively (*p* < 0.01), accompanied by a 1.2-fold upregulation of major capsid protein (MCP) expression compared to untreated infected controls (Figure 6A). Conversely, liproxstatin-1 treatment reduced intracellular viral DNA load by 30.6% (*p* < 0.01) and extracellular virion production by 77.7% (*p* < 0.01), with a corresponding 48.1% decrease in MCP expression (Figure 6B). These results suggested that viral propagation is significantly enhanced by ferroptosis while being suppressed by ferroptosis inhibitors.

### 3.3. ISKNV Triggers Ferroptosis Through Suppressing GPx4 and Promoting ACSL4

**Figure 7:**
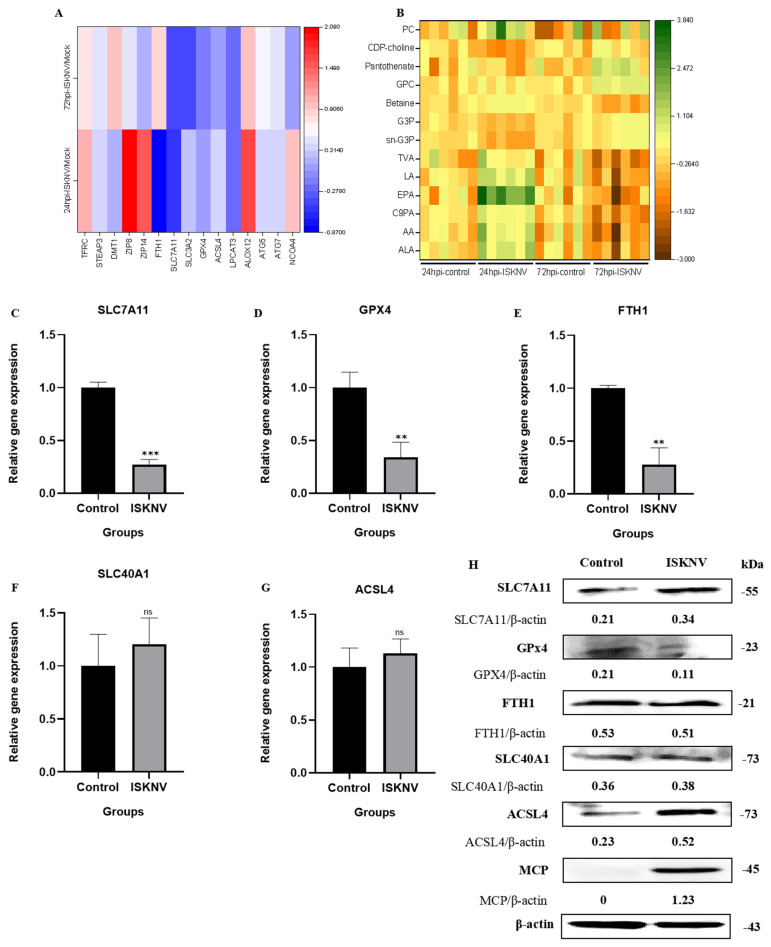
ISKNV induces ferroptosis by suppressing GPx4 and promoting ACSL4. (**A**) Heat map of the mRNA level involved in ferroptosis in CPB cells infected with ISKNV at 24 hpi and 72 hpi. Each column represents one sample. (Transferrin receptor (TFRC), Six-Transmembrane Epithelial Antigen of Prostate 3 (STEAP3), Zinc- and iron-related protein 8 (ZIP8), Zrt- and Irt-like protein 14 (ZIP14), Divalent metal transporter 1 (DMT1), Ferritin heavy chain (FTH1), Solute carrier family 7 member 11 (SLC7A11), Solute carrier family 3 member 2 (SLC3A2), Glutathione peroxidase 4 (GPx4), Acyl-CoA synthetase long-chain family member 4 (ACSL4), Arachidonate 12-Lipoxygenase (ALOX12), Lysophosphatidylcholine acyltransferase 3 (LPCAT3), Autophagy-related protein 5 (ATG5), Autophagy-related protein 7 (ATG7), and Nuclear receptor coactivator 4 (NCOA4).) (**B**) Heat map of differentially expressed fatty acids and phospholipids in CPB cells infected with ISKNV at 24 hpi and 72 hpi. Each column represents one sample. (Alpha-Linolenic acid (ALA), Arachidonic Acid (AA), Cis-9-Palmitoleic acid (C9PA), Eicosapentaenoic acid (EPA), Linoleic acid (LA), Trans-Vaccenic acid (TVA), sn-Glycerol 3-phosphoethanolamine (sn-G3P), Glycerophosphocholine (GPC), Glycerol 3-phosphate (G3P), Betaine, Pantothenate, Cytidine 5′-diphosphocholine (CDP-choline), and Phosphatidylcholine (PC)). (**C**–**G**) The mRNA expression of *SLC7A11*, *GPx4*, *FTH1*, *SLC40A1*, and *ACSL4* in CPB cells infected with ISKNV at 72 hpi, respectively. ** *p* < 0.01, and *** *p* < 0.001, with *p* > 0.05 considered not significant (ns). (**H**) The protein expression of SLC7A11, GPx4, FTH1, SLC40A1, ACSL4, and MCP in CPB cells infected with ISKNV at 72 hpi, respectively.

Transcriptomic profiling of ISKNV-infected CPB cells revealed dynamic regulation of ferroptosis-associated pathways at 24 hpi and 72 hpi, including iron metabolism genes (TFRC, STEAP3, ZIP8, ZIP14, DMT1, and FTH1), xCT-/GPx4 axis genes (SLC7A11, SLC3A2, and GPx4), and lipid metabolism genes (ACSL4, ALOX12, and LPCAT3), as well as ATG5/7-NCOA4 pathway components (ATG5, ATG7, and NCOA4) (Figure 7A). Metabolomic analysis identified time-dependent lipid alterations, with polyunsaturated fatty acids (ALA, AA, C9PA, EPA, LA, and TVA), phospholipid synthesis precursors (sn-G3P and GPC) and associated metabolites (G3P, betaine, pantothenate, CDP-choline, and PC) suggesting staged lipid peroxidation dynamics (Figure 7B). Subsequently, qRT-PCR validation confirmed marked downregulation of *SLC7A11*, *GPx4*, and *FTH1* at 72 hpi, while *SLC40A1* and *ACSL4* showed non-significant upward trends (Figure 7C–G). Western blot analysis revealed corresponding protein-level changes to confirm that GPx4 was significantly suppressed, ACSL4 induced, and SLC7A11 moderately upregulated, with FTH1 and SLC40A1 remaining stable (Figure 7H). These results suggest that GPx4 and ACSL4 may play a key role in ISKNV-induced ferroptosis in CPB cells.

## 4. Discussion

Ferroptosis is a recently discovered form of programmed cell death, and numerous studies have shown that ferroptosis plays a role in the development and progression of various diseases, including neurological disorders and cardiovascular diseases [25]. Notably, recent advancements have revealed viruses exploit ferroptotic pathways to facilitate their replication or trigger ferroptosis as a cytopathic mechanism; for example, hepatitis B virus protein X stabilizes enhancer of zeste homolog 2 (EZH2) and promotes trimethylation of H3K27, which inhibits SLC7A11 and induces ferroptosis in acute liver failure [26], while herpes simplex virus (HSV) leads to enhanced oxidative stress, decreases GSH concentration in host cells, and induces lipid peroxidation to produce highly reactive membrane lipid hydroperoxides [27]. The hallmarks of ferroptosis include mitochondrial damage, iron homeostasis imbalance, iron-dependent ROS production, and lipid peroxidation [6,28]. Our findings demonstrated that ISKNV infection in CPB cells induced characteristic morphological and biochemical alterations consistent with ferroptotic cell death. The observed mitochondrial pathology—including marked shrinkage, increased membrane density, and progressive cristae reduction—represents the hallmark ultrastructural signature of ferroptosis. The biochemical profile of ISKNV-infected cells exhibited the accumulation of Fe^2+^, ROS, and MDA. These morphological and biochemical alterations have been previously documented in other viral systems [29,30].

Some studies have demonstrated that viruses exploit ferroptotic pathways to facilitate their replication. Xia et al. revealed that the ferroptosis inhibitor inhibited murine hepatitis virus strain A59 (MHV-A59) propagation, inflammatory cytokine release, and cell syncytia formation in primary macrophages [31]. Similarly, Cheng et al. reported that H1N1 swine influenza virus (SIV) induced ferroptosis in host cells, and that ferroptosis inhibition using ferrostatin-1 not only reduced viral replication but also mitigated inflammatory responses [32]. In the present study, we also found ferroptosis inhibition with liproxstatin-1 suppressed ISKNV yield. Furthermore, it was interesting that ferroptosis induction with erastin enhanced ISKNV replication. Erastin induced ferroptosis through interfering with multiple sites, including voltage-dependent anion-selective channels, cystine/glutamate exchange transporters, and glutathione peroxidase 4 [33]. Cystine/glutamate exchange transporters, designated to the system *Xc-*, uptake cystine and release Glu in a 1:1 ratio [34]. Our previous study revealed ISKNV replication required glutamine [22]. Therefore, we hypothesized that the addition of an erastin-inhibiting system *Xc-* led to an increase in intracellular glutamate levels, thereby facilitating ISKNV replication.

Japanese encephalitis virus (JEV) infection led to neuronal ferroptosis by inhibiting GPx4 function and upregulating ACSL4 expression, resulting in neuronal damage and inflammation, and inhibiting ferroptosis was shown to reduce the mortality rate and alleviate neuroinflammation and brain damage in a mouse model [29]. GPx4 is the only enzyme in the cell that is capable of directly reducing lipid peroxides and plays a core role in inhibiting lipid peroxidation and preventing ferroptosis [35]. ACSL4 specifically catalyzes the conversion of polyunsaturated fatty acids such as AA and AdA into their corresponding acyl-CoA derivatives, providing substrates for phospholipid synthesis. This, in turn, promotes the accumulation of phospholipids containing polyunsaturated fatty acids (PUFA-PLs) on the cell membrane [36]. PUFA-PLs are the primary substrates for lipid peroxidation in ferroptosis, and their accumulation on the cell membrane makes the membrane more susceptible to oxidative stress [37]. Our results showed that after 72 h of ISKNV infection in CPB cells, GPx4 showed a significant reduction at both the transcription and protein levels, and ACSL4 was significantly upregulated. In the metabolomic analysis, polyunsaturated fatty acids and phospholipids were increased in ISKNV-infected CPB cells. These findings demonstrated that ISKNV infection induced ferroptosis in CPB cells through downregulation of the antioxidant enzyme GPx4 and upregulation of ACSL4.

In conclusion, it is the first study to demonstrate that ISKNV induces ferroptosis in CPB cells and ferroptosis promotes ISKNV replication. Notably, ISKNV infection induced ferroptosis through downregulation of the antioxidant enzyme GPx4 and upregulation of ACSL4. These findings significantly advance our understanding of ISKNV pathogenesis.

## Figures and Tables

**Table 1:** Primers for detection of genes.

Primer Name	Sequence (5′-3′)	Amplicon Size (bp)	Accession Number
18S-F	CATTCGTATTGTGCCGCTAGA	120	XR_006376550.1
18S-R	CAAATGCTTTCGCTTTGGTC		
SLC7A11-F	GAGGAGGTAGATAACCCTGAACGG	120	XM_044205657.1
SLC7A11-R	CTCCTCTGCTGACATCACAGTG		
GPX4-F	CAACAGATGATCCCAGCGTGGT	120	XM_044218327.1
GPX4-R	CACGCACACCAATACCCTGAAG		
FTH1-F	CGCTGTGACGCTGATAATTATCC	126	XM_044194482.1
FTH1-R	CTGCAGTTGATTGACAACTAGC		
SLC40A1-F	CTAACCCACTCTGAGATTGTACGG	125	NC_058053.1
SLC40A1-R	CTGGTACAGTTCATGTGGTGCTG		
ACSL4-F	GCGTAAGCCTCAGCTATTCCAG	132	XM_044206073.1
ACSL4-R	GGGAACAAACAGCGTTTCTTCAAC		

**Table 2:** The primers for detection of virus copy number.

Primer Name	Sequence (5′-3′)	Amplicon Size	Reference
ISKNV-F	CGAGGCCACATCCAACATC	85 (bp)	Ma B. F. [24]
ISKNV-R	CGCCTTTAACGTGGGATATATTG		
ISKNV-Probe	CACCAAACTGACCGCGGACTCGT		

## Data Availability

The data that support the findings of this study are available from the corresponding author upon reasonable request.
